# Numerical Study of Step Drill Structure on Machining Damage in Drilling of CFRP/Ti Stacks

**DOI:** 10.3390/ma16176039

**Published:** 2023-09-02

**Authors:** Chen Chen, Qing Zhao, Aixu Wang, Jing Zhang, Qing Qu, Zhanli Shi

**Affiliations:** Naval Architecture and Ocean Engineering College, Dalian Maritime University, Dalian 116026, China; 1120211815@dlmu.edu.cn (Q.Z.); wax1808@dlmu.edu.cn (A.W.); zjing@dlmu.edu.cn (J.Z.); qqing1559@dlmu.edu.cn (Q.Q.)

**Keywords:** CFRP/Ti stacks, step drill, tool structure, simulation, drilling damage

## Abstract

The tool structure is an important factor affecting the damage of CFRP/Ti stacks machining. However, the impact of tool structure on the formation process of stacks hole damage cannot be fully revealed through experimental methods alone. In contrast, finite element simulation can effectively overcome the limitations of experiments. In this study, a numerical simulation model is established to investigate the relationship between step drill structure and formation process of CFRP/Ti stacks hole damage. Based on this, the research discusses the effect of step drill structure on the burr height of Ti layer, delamination of CFRP, aperture deviation, defects in hole surface. The results show that when the stacking sequence is CFRP to Ti, the burr height of Ti at hole exit decreases first and then increases with the rising of the ratio of primary drill bit diameter to secondary drill bit diameter (*k_d_*). When *k_d_* is 0.6, the burr height of Ti at hole exit is the lower. As *k_d_* increasing from 0.4 to 1.0, delamination factor of CFRP increases by 2.57%, which are affected little by the step drill structure due to the support of Ti. Besides, the aperture size deviation decreases first then increases with the rising of *k_d_*, and the minimum aperture size deviation is 2.09 μm when *k_d_* is 0.6. In addition, as *k_d_* is 0.6, the hole wall defect is fewer. In conclusion, step drill with *k_d_* of 0.6 is suitable for drilling of CFRP/Ti stacks.

## 1. Introduction

Carbon fiber reinforced plastic (CFRP) has been widely used in aerospace, automotive and other fields due to its outstanding advantages such as high temperature resistance, high specific strength and corrosion resistance [1,2]. However, due to the low-strength in transverse direction, CFRP cannot be applied to suffer large bending loads. In addition, the titanium alloy (Ti) can bear high compressive and tensile strength. CFRP and Ti are mutually coordinated in physical and mechanical properties, which can cover the shortcomings of using a single material and improve the reliability of the structure. Therefore, CFRP/Ti stacks can meet the performance and lightweight requirements of large aircraft structural components under extreme service conditions [3,4,5].

In practical applications, CFRP/Ti stacks are often connected and assembled through bolt connections and riveting [6]. Therefore, drilling is the most commonly used processing method for CFRP/Ti stacks, and the quality of hole machining can seriously affect the assembly quality of stacks structural components [7]. However, both CFRP and Ti are difficult to machine materials in practice, and there are significant differences in their material and processing properties, which makes stacks prone to serious hole damage defects, affecting the quality of drilling and ultimately affecting the performance of components [8].

The tool structure and angle directly affect the contact state between the tool and the material, thereby affecting drilling damage. In order to effectively suppress the drilling damage of CFRP/Ti stacks, extensive research have been conducted on the relationship between tool angle and machining damage. Jia et al. [9] proposed a novel small point angle special drill bit structure suitable for drilling CFRP, which has a great effect in effectively reducing the exit damage of CFRP. SenthilKumar et al. [10] found that there is a great correlation between the point angle of drills and machining quality. And through experimental comparison, it was found that compared with the drills with point angle of 118°, the drills with 130° point angle have better chip removal performance and are less prone to tool wear. Shi et al. [11] found that there is a significant difference in the point angle of drill bits suitable for drilling CFRP and Ti. Through experiments, it was found that selecting a smaller point angle to drill CFRP can effectively avoid delamination damage at the exit of CFRP, while selecting a larger point angle for drilling Ti can effectively reduce burrs on exit.

When drilling of CFRP/Ti stacks, a series of machining damages may occur, most of which are closely related to the factor of thrust force, which is also closely related to the tool structure. Therefore, selecting a tool with appropriate geometric structure is particularly important. Baon et al. [12] found that double point angle tool is suitable for drilling CFRP, but is not suitable for drilling Ti and CFRP/Ti stacks. Liu et al. [13] proposed that the plane rake–faced twist drill is suitable for drilling CFRP, it was found that compared with the ordinary twist drill, thrust force generated by the plane rake–faced twist drill is smaller, which can effectively inhibit the hole machining damage. Alonso et al. [14] conducted drilling experiments on CFRP/Ti stacks using step drill and found that step drill has a certain effect in reducing thrust force, thereby effectively reducing drilling damage and achieving good hole quality. Wang et al. [15] pointed out that when using step structures for drilling, the primary cutting edge of the step structures also has a certain effect in reducing thrust force, thereby effectively reducing the drilling damage of CFRP. Brinksmeier et al. [16] also found that the stepped twist drill with special structure is more suitable for drilling stacks materials by comparing the cutting performance of carbide tools with different geometric shapes. Leng et al. [17,18] compared CFRP and CFRP/Ti stacks materials processed with step drill and twist drill, and found that the drilling thrust force generated by step drill is about half of that of twist drill, and better aperture accuracy and surface roughness can be obtained by drilling with step drill.

The above experimental research can obtain the effect of tool angle and tool structure on drilling damage of stacks. However, as the tool structure is complex, it is difficult to effectively reveal the interaction process between tools and materials through experimental methods alone. The numerical simulation method can overcome this shortcoming, and can be used to further explore the impact of tool structure on the drilling damage of stacks. Diaz-Alvarez et al. [19] investigated the relationship between tool point angle and composite drilling damage through numerical simulation, and verified the correctness of the established numerical simulation results through experiments. Wang et al. [20] established a drilling simulation model for CFRP/Al stacks using finite element analysis software, and compared and analyzed the relationship between tool structure and interlayer delamination damage of CFRP. Isbilir et al. [21] established numerical models for drilling CFRP with different tools under the same processing parameters, and found that compared with ordinary twists drills, step drills produce less thrust force and torque, which can suppress the delamination damage of CFRP.

In summary, compared with twist drills, step drills are more suitable for drilling of CFRP/Ti stacks. While, the effects of the structure of step drill on drilling damage of stacks are still need to research furtherly. On account of that the finite element simulation method can be used to investigate the influence of tool structure effectively. Therefore, in this study, numerical models are developed to simulate the complete drilling process of CFRP/Ti stacks with different step drills. Through this model, the effect of the ratio of primary drill bit diameter to secondary drill bit diameter (*k_d_*) on the burr height of Ti layer, delamination of CFRP, aperture deviation, defects in hole surface are obtained. This study can provide the basis for optimization of the tool structure for drilling of CFRP/Ti stacks.

## 2. Numerical Setup

### 2.1. Modeling for the CFRP

According to the three-dimensional generalized Hooke’s law, under the symmetric condition of orthogonality and anisotropy, the mechanical properties of CFRP laminates can be described as follows.

(1) {σ}=[C]{ε}

where {*σ*} and {*ε*} respectively is the stress vector and the strain vector respectively, [*C*] signifies the stiffness matrix. And the equation can be expanded as follows.

(2) $$\left\{\begin{array}{c} \sigma_{11}\\ \sigma_{22}\\ \sigma_{33}\\ \sigma_{12}\\ \sigma_{23}\\ \sigma_{31} \end{array}\right\}=\left[\begin{array}{cccccc} C_{11} & C_{12} & C_{13} & 0 & 0 & 0\\ C_{21} & C_{22} & C_{23} & 0 & 0 & 0\\ C_{31} & C_{32} & C_{33} & 0 & 0 & 0\\ 0 & 0 & 0 & C_{44} & 0 & 0\\ 0 & 0 & 0 & 0 & C_{55} & 0\\ 0 & 0 & 0 & 0 & 0 & C_{66} \end{array}\right]\left\{\begin{array}{c} \varepsilon_{11}\\ \varepsilon_{22}\\ \varepsilon_{33}\\ \varepsilon_{12}\\ \varepsilon_{23}\\ \varepsilon_{31} \end{array}\right\}$$

where the expressions for the stiffness coefficient in [*C*] are as follows.

(3) $$C_{11}=\Delta E_{1}(1-\nu_{23}\nu_{32})$$

(4) $$C_{22}=\Delta E_{2}(1-\nu_{13}\nu_{31})$$

(5) $$C_{33}=\Delta E_{3}(1-\nu_{12}\nu_{21})$$

(6) $$C_{12}=\Delta E_{2}(\nu_{12}+\nu_{13}\nu_{32})=\Delta E_{1}(\nu_{21}+\nu_{31}\nu_{23})$$

(7) $$C_{13}=\Delta E_{3}(\nu_{13}+\nu_{12}\nu_{23})=\Delta E_{1}(\nu_{31}+\nu_{21}\nu_{32})$$

(8) $$C_{23}=\Delta E_{2}(\nu_{23}+\nu_{21}\nu_{13})=\Delta E_{3}(\nu_{32}+\nu_{12}\nu_{31})$$

(9) $$C_{44}=C_{23},C_{55}=C_{31},C_{66}=C_{12}$$

(10) $$\Delta =\frac{1}{1-\nu_{12}\nu_{21}-\nu_{23}\nu_{32}-\nu_{13}\nu_{31}-2\nu_{12}\nu_{23}\nu_{31}}$$

Due to the good consistency between its theoretical and experimental values, in this paper, the three-dimensional Hashin failure criterion is used to predict material damage in CFRP. Different forms of failure identification methods are used separately [22].

Fiber tensile failure (*σ*_11_ ≥ 0)

(11) $$F_{ft}={\left(\frac{\sigma_{11}}{X_{t}}\right)}^{2}+{\left(\frac{\tau_{12}}{S_{12}}\right)}^{2}+{\left(\frac{\tau_{13}}{S_{13}}\right)}^{2}$$

Fiber compression failure (*σ*_11_ < 0)

(12) $$F_{fc}={\left(\frac{\sigma_{11}}{X_{c}}\right)}^{2}$$

Matrix tensile failure (*σ*_22_ + *σ*_33_ ≥ 0)

(13) $$F_{mt}={\left(\frac{\sigma_{22}+\sigma_{33}}{Y_{t}}\right)}^{2}+\frac{\left(\tau_{23}^{2}-\sigma_{22}\sigma_{33}\right)}{S_{23}^{2}}+{\left(\frac{\tau_{12}}{S_{12}}\right)}^{2}+{\left(\frac{\tau_{13}}{S_{13}}\right)}^{2}$$

Matrix compression failure (*σ*_22_ + *σ*_33_ < 0)

(14) $$F_{mc}=\frac{1}{Y_{c}}\left[{\left(\frac{Y_{c}}{2S_{12}}\right)}^{2}-1\right]\left(\sigma_{22}+\sigma_{33}\right)+{\left(\frac{\sigma_{22}+\sigma_{33}}{2S_{12}}\right)}^{2}+\frac{\left(\tau_{23}^{2}-\sigma_{22}\sigma_{33}\right)}{S_{23}^{2}}+{\left(\frac{\tau_{12}}{S_{12}}\right)}^{2}+{\left(\frac{\tau_{13}}{S_{13}}\right)}^{2}$$

Tensile delamination failure (*σ*_33_ ≥ 0)

(15) $$F_{dt}={\left(\frac{\sigma_{33}}{Z_{t}}\right)}^{2}+{\left(\frac{\tau_{13}}{S_{13}}\right)}^{2}+{\left(\frac{\tau_{23}}{S_{23}}\right)}^{2}$$

Shear delamination failure (*σ*_33_ < 0)

(16) $$F_{dc}={\left(\frac{\tau_{13}}{S_{13}}\right)}^{2}+{\left(\frac{\tau_{23}}{S_{23}}\right)}^{2}$$

where *F_fi_*, *F_mi_*, and *F_di_* (*I* = *t, c*) in the discriminant are all failure factors. When their values are greater than 1, the material failure damage occurs. The mechanical properties of CFRP used in this study are derived from reference [23] and summarized in Table 1.

### 2.2. Modeling for the Titanium Alloy

In order to more accurately simulate the entire process of material removal during the machining of Ti, this model can be defined using the Johnson-Cook (JC) constitutive model and damage criteria for Ti. The expression of the JC constitutive model defining the constitutive equation of Ti is as follows [24].

(17) $$\sigma =\left(A+B\varepsilon^{n}\right)\left[1+C\ln\left(\frac{\dot{\varepsilon }}{{\dot{\varepsilon }}_{0}}\right)\right]\left[1-{\left(\frac{T-T_{r}}{T_{m}-T_{r}}\right)}^{m}\right]$$

where *σ* represents the equivalent stress; *A* and *B* is the yield strength and stress hardening parameter, respectively; *ε* represents the equivalent plastic strain; *n* is the hardening index; *C* represents the strain rate strengthening parameter; *ε* and $\dot{\varepsilon_{0}}$ respectively represent the equivalent plastic strain and the reference equivalent plastic strain rate; *T_m_* and *T_r_* represent melting temperature and ambient temperature, respectively; and *m* is thermal softening parameter.

In addition, the expression of JC damage criteria is as follows [25].

(18) $$D=\sum \frac{\Delta \varepsilon_{f}}{{\bar{\varepsilon }}_{f}}$$

(19) $$\varepsilon_{f}=\left[D_{1}+D_{2}\exp\left(-\alpha D_{3}\right)\right](1+D_{4}\ln\frac{\dot{\varepsilon }}{{\dot{\varepsilon }}_{0}})\left[1+D_{5}\frac{T-T_{0}}{T_{melt}-T_{0}}\right]$$

where *D* represents the failure parameter, Δ*ε_f_* and ${\bar{\varepsilon }}_{f}$ respectively represent the equivalent plastic strain increment and the equivalent plastic strain at damage initiation, *α* is obtained by the ratio of hydrostatic pressure to Mises stress, representing the stress. The parameters of the JC constitutive model and damage criteria of the Ti are list in Table 2.

### 2.3. Modeling for the Interface Layer

In practical applications, there may be a certain thickness gap between CFRP layer and Ti layer, so it is necessary to establish an interface layer in the simulation model to simulate this gap. The constitutive behavior of the interface is defined by the traction-separation law, and the constitutive behavior can be described as follows [26].

(20) $$\left\{\begin{array}{c} \sigma_{n}\\ \sigma_{s}\\ \sigma_{t} \end{array}\right\}=\left[\begin{array}{ccc} \left(1-D\right)K_{nn} & 0 & 0\\ 0 & \left(1-D\right)K_{ss} & 0\\ 0 & 0 & \left(1-D\right)K_{tt} \end{array}\right]\left\{\begin{array}{c} \varepsilon_{n}\\ \varepsilon_{s}\\ \varepsilon_{t} \end{array}\right\}$$

(21) $$\varepsilon_{n}=\frac{\delta_{n}}{T_{c}},\varepsilon_{s}=\frac{\delta_{s}}{T_{c}},\varepsilon_{t}=\frac{\delta_{t}}{T_{c}}$$

where *σ_n_*, *σ_s_* and *σ_t_* are the stresses; *ε_n_*, *ε_s_* and *ε_t_* are the strains; *K_nn_*, *K_ss_* and *K_tt_* is the stiffness; *δ_n_*, *δ_s_* and *δ_t_* are the displacements; *T_c_* represents the thickness of the interface.

The Cohesive interface element damage criteria is based on the quadratic stress criteria, and the formula is as follows [26].

(22) $${\left(\frac{\sigma_{n}}{t_{n}^{f}}\right)}^{2}+{\left(\frac{\sigma_{s}}{t_{s}^{f}}\right)}^{2}+{\left(\frac{\sigma_{t}}{t_{t}^{f}}\right)}^{2}=1$$

where $t_{n}^{f}$, $t_{s}^{f}$ and $t_{t}^{f}$ represent the strength.

The parameters related to the interface layer material of stacks structure are selected by referring to the relevant literature [26,27], and the specific parameters are list in Table 3.

### 2.4. Geometrical Modelling for Drilling of Stacks

**Figure 1:**
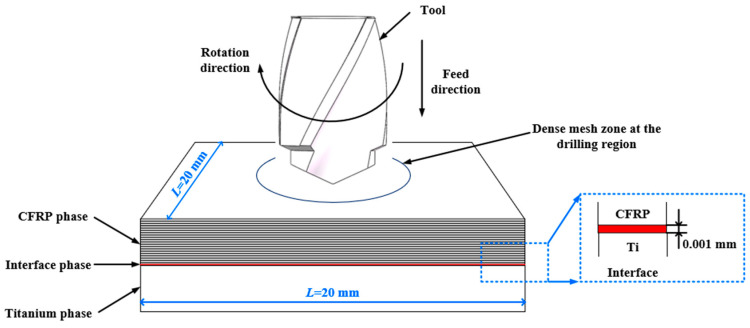
Drilling simulation model for CFRP/Ti stacks.

Figure 1 is a schematic diagram of the simulation model for drilling of CFRP/Ti stacks using step drill. The size of the model is 20 mm × 20 mm × 8 mm. The geometric parameters of the step tool are list in Table 4. The CFRP is made up of 20 layers with the thickness of each layer is 0.2 mm. To eliminate coupling effects and improve the effective stiffness and stability of laminated plates, the ply direction of multi-directional CFRP laminates is [(−45°/0°/45°/90°)_2_/0°/0°]_S_.

The CFRP layer and the Ti layer are both controlled by the 8-node linear brick element (C3D8R), reduced integration, and hourglass enhancement control. The interface layer uses the 8-node three-dimensional cohesive element (COH3D8), and the whole element is also controlled by hourglass enhancement. Since there is no need to consider issues such as tool wear and deformation, rigid body constraints are set on the tool, and the 4-node linear tetrahedron element (C3D4) is used as the mesh element type.

### 2.5. Experiment Validation of CFRP/Ti Stacks Drilling Model

**Figure 2:**
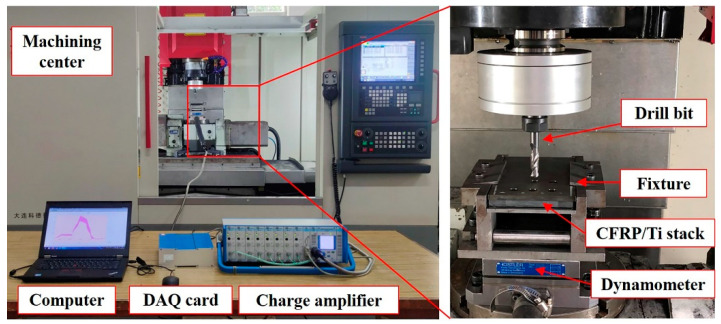
Experiment setup of drilling tests.

The correctness of the simulation model is verified by drilling experiments on the five-axis CNC machining center in this study. The experimental workpieces are placed in a special fixture and connected to the dynamometer, which is fixed on the worktable, and the experiment setup is shown in Figure 2. The thickness of the Ti plate is 4 mm. The thickness of the multidirectional CFRP laminates is 4 mm, and the layer direction is [(−45°/0°/45°/90°)_2_/0°/0°]_S_, which is same with that in the simulation model. The tools used in the experiment are step drills, and the geometrical parameters of the step drill are same with those in the simulation as list in Table 4.

All the trials are carried out in dry cutting conditions with a constant drilling parameters of spindle speed of 600 rpm and feed rate of 0.06 mm/rev. A new step drill is used in each group for drilling 3 holes. After drilling process, the three-dimensional morphology of burrs of the Ti layer at the hole exit are measured by Alicona Infinite Focus G5 optical measurement system, and base on the three-dimensional morphology, the two-dimensional contour and height value of the titanium alloy exit burr are extracted 4 points on the circumference with an interval of 90° are selected as the measurement positions, and the degree of burr damage at the exit of the titanium alloy are evaluated based on the average value of the burr height at the selected measurement positions.

**Figure 3:**
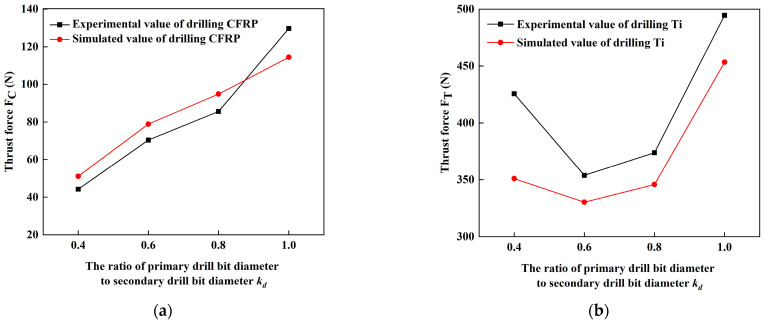
Comparison between simulated and experimental value of thrust force. (**a**) Thrust force of stable drilling of CFRP; (**b**) Thrust force of stable drilling of Ti.

Thrust force is a key factor reflecting the relationship between workpieces and tools in the drilling process, therefore, in this study, thrust force is selected as an indicator to verify the simulation model. The comparison of thrust force between the simulated and experimental value in the stable drilling of CFRP and Ti are shown in Figure 3. From Figure 3, it can be seen that when the stacking sequence is CFRP to Ti, with the rising of *k_d_*, thrust force value in the stable section of the drilling CFRP laminated plate increases accordingly, and thrust force in the stable section of the drilling Ti first decreases and then increases.

Based on the results, it can be known that the overall variation trend of thrust force obtained from the simulation and experimental measurements is in the same variation pattern and good consistency. When *k_d_* is 0.4, the error between the simulated and experimental value of thrust force in the stable zone of the drilling CFRP layer reaches the maximum value of 15.59%. Although the error exceeds 10%, it is less than 20%, which is within a reasonable error range. Therefore, the predicted thrust force value is close to the experimental measurement value, which can verify the correctness of the established finite element simulation model.

**Figure 4:**
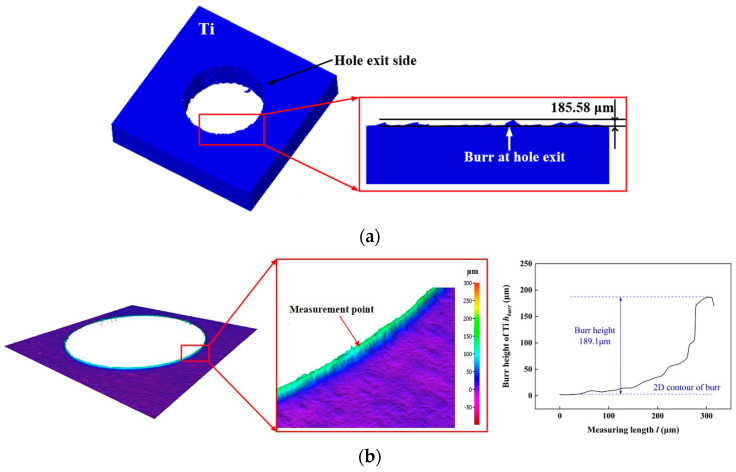

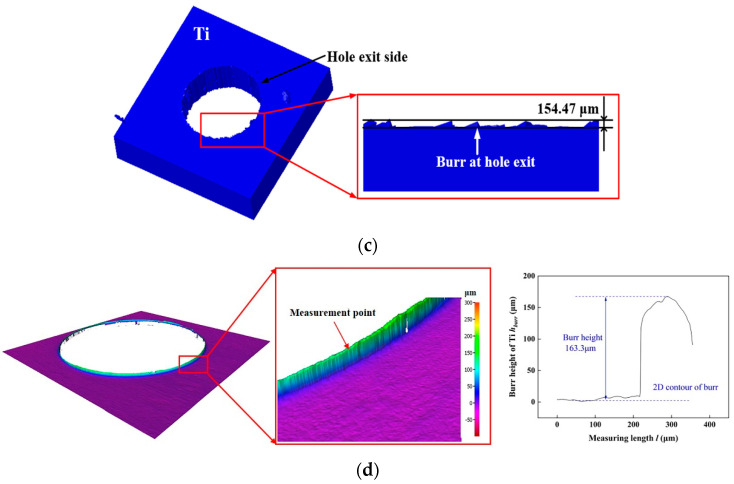
Simulated and experimental measured morphology of Ti at hole exit. (**a**) The simulated morphology of Ti at hole exit as *k_d_* is 0.4; (**b**) Experimental measurement morphology of Ti at hole exit as *k_d_* is 0.4; (**c**) The simulated morphology of Ti at hole exit as *k_d_* is 0.6; (**d**) Experimental measurement morphology of Ti at hole exit as *k_d_* is 0.6.

In addition, the burrs of Ti layer is a serious damage as drilling of CFRP/Ti stacks. The morphology of burrs of the titanium alloy layer simulated and experimental measured are shown in Figure 4. Both simulation and experiment have observed that the burrs of Ti layer occurs around the hole exit. In this study, the maximum height of burrs is used as an another indicator to verify the simulation model. When *k_d_* is 0.4, the maximum value of burrs height simulated is 185.58 μm. The maximum value of burrs measured in the experiment is 189.10 μm. And the error of the maximum height of the burr is about 1.86%. Besides, when *k_d_* is 0.6, the maximum values of burrs height simulated and experimental measured are 154.47 μm and 148.94 μm, and the maximum error is about 3.71%. The above results indicate that the burr height simulated has a small error compared to the experimental results. Based on these results, it can be seen that the height of burrs at hole exit of Ti obtained from simulation and experimental measurements have good consistency. The results of thrust force and burrs of Ti layer can effectively prove the reliability of the CFRP/Ti stacks drilling model.

## 3. Numerical Result and Discussion

### 3.1. Effect of Step Drill Structure on the Titanium Alloy Burr at Hole Exit

**Figure 5:**
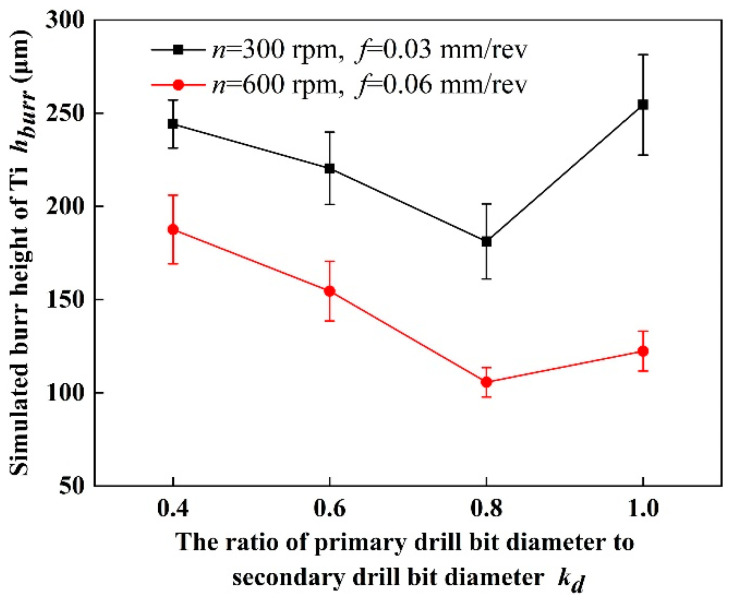
Effect of step drill structure on the burr height of Ti at hole exit.

Based on the simulation model, the burr height of Ti at hole exit as drilling stacks with four kinds of step drills under two drilling parameters are obtained and shown in Figure 5. Based on the results, it can be learned that when the stacking sequence is CFRP to Ti, the height of the titanium alloy burr at hole exit decreases first and then increases with the rising of *k_d_*. When *k_d_* is 0.6, the height of the titanium alloy burr at hole exit is the lower.

As drilling of the titanium alloy layer, the material suffers the extrusion deformation of the cutting edge, a portion of the material is carried away from the surface of the workpiece along the direction of motion with the shear action of the tool, while another portion of the residual material remains on the surface of the Ti, which forms the burrs. When drilling Ti layer with step drills, primary drill bit acts as a pre-drilled hole on the workpiece, and the secondary cutting edge will removed the burrs generated by the primary cutting edge. Therefore, using step drill can effectively reduce the burr height of Ti at hole exit.

And as *k_d_* increases, the size of primary drill bit increases and the size of the second cutting edge acting on the remaining material decreases, which could cause the extrusion stress acting on the remaining material decrease and more material can be removed before deformation occurring. Hence the height of burrs could decreases first as *k_d_* increases. While, as *k_d_* increases furtherly, the size of primary drill bit is large, and serious burrs of Ti could occur during the drilling process by the primary drill bit, meanwhile, the secondary cutting edge of the tool could not remove the burrs. In addition, we have learned from the previous discussion that when *k_d_* exceeds 0.8, thrust force of the drill bit increases with the increase of *k_d_*. At this point, the remaining material at the titanium alloy exit cannot resist thrust force of the drill bit, resulting in plastic deformation of the remaining material under the squeezing effect of the tool and the formation of exit burrs. Hence, the burrs height of Ti then increases with the increases of *k_d_*, when *k_d_* exceeds 0.8. However, as *k_d_* is 0.4, the primary drill bit is too small, the second cutting edge of the tool will remove the burr generated by the primary cutting edge and then re-extrude the remaining material to produce burr. So the height of the titanium alloy burr at hole exit is relatively high.

### 3.2. Effect of Step Drill Structure on the Delamination Damage of CFRP

**Figure 6:**
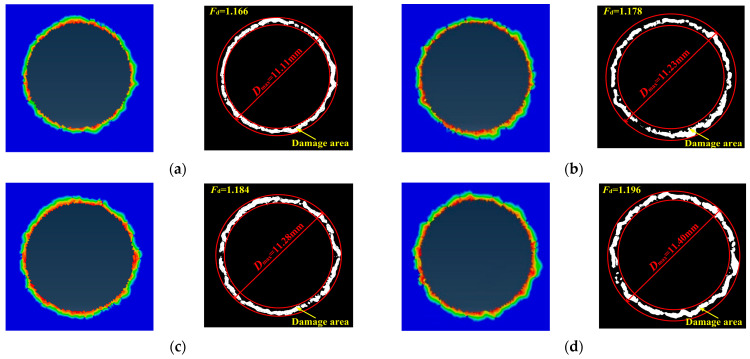
Morphology of CFRP hole exit. (**a**) *k_d_* is 0.4; (**b**) *k_d_* is 0.6; (**c**) *k_d_* is 0.8; (**d**) *k_d_* is 1.0.

In this study, in order to investigate the effect of step drill structure parameters on the delamination damage at the exit of CFRP laminates in stacks, delamination factor *F*_d_ is used to quantitatively analyzed the degree of delamination damage. *F*_d_ is defined as the ratio of the diameter of the damaged area *D*_max_ to the nominal diameter of the hole *D*_0_. The simulation results are obtained using ABAQUS finite element software. As shown in the left figure of Figure 6a, the colored area around the hole is the delamination damage area at the exit of the CFRP hole. Based on the simulation results, the damage area of CFRP hole exit are extracted through the program user defined. The white area shown in the right figure of Figure 6a is the delamination damage area at the outlet of the CFRP hole. Figure 6 shows the exit morphology of CFRP holes obtained by drilling of CFRP/Ti stacks with four types of step drills.

According to the results of *F*_d_, it can be known that as *k_d_* increases, *F*_d_ slightly increases. This is because step drills can simultaneously reduce the volume of drilling material and disperse thrust force generated by the drilling material. From Figure 3, it is obvious that with the rising of *k_d_*, thrust force value of the drilling stationary section increases accordingly. The delamination damage defect at the exit of CFRP is greatly affected by drilling thrust force. When drilling stacks with step drill, primary drill bit acts as a pre-drilled hole on the workpiece, and the secondary cutting edge will remove part of the delamination caused by primary drill bit, thereby reducing delamination. However, when the stacking sequence is CFRP to Ti, due to the support of Ti under the CFRP, delamination damage factor of CFRP only increase by 2.57% as *k_d_* increases from 0.4 to 1.0. Therefore, the support of the titanium alloy layer can offset part of the punching on the CFRP by the step drill. Hence, delamination of CFRP as drilling of CFRP/Ti stacks is less affected by the change of *k_d_*.

### 3.3. Effect of Step Drill Structure on the Aperture Size

Due to the significant differences in physical and mechanical properties between CFRP and Ti, the problem of inconsistent aperture size between the CFRP and Ti often occurs during the hole machining process of CFRP/Ti stacks, which greatly affects assembly accuracy and may lead to failure of the component connection parts.

**Figure 7:**
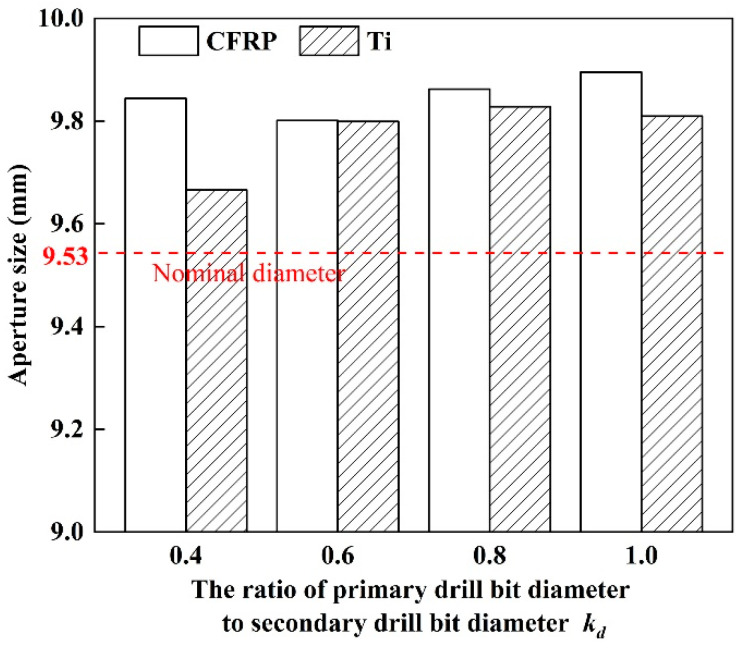
Aperture size obtained from CFRP/Ti stacks drilling simulation model.

The simulated aperture size of stacks as drilling with different step drills are as shown in Figure 7. Based on the results, it can be obtained that the aperture size deviation of stacks drilled by four types of step drills are 178.31 μm, 2.09 μm,34.34 μm and 85.5 μm, respectively. When *k_d_* is 0.6, the aperture size deviation is the smallest and the consistency of the aperture size is the best.

From Figure 7, it can be seen that the aperture size of the CFRP is larger than that of Ti. This is because the elastic modulus of Ti is smaller than that of CFRP, which can result in a larger springback in drilling process. Hence, the deviation of aperture size of Ti to the nominal diameter is smaller than that of CFRP layer. Meanwhile, due to the stacking sequence is CFRP to Ti, the chips generated by the tool drilling the titanium alloy may scratch the hole wall of CFRP layer, which leads to an increase in the aperture size of CFRP layer.

Thrust force is the main factor affecting aperture size. As thrust force is reduced, the vibration during the drilling process will also be reduced, which is beneficial for suppressing the deviation of aperture size. Combined with the results of thrust force with *k_d_* in Figure 3, it can be learned that drilling of stacks with step drills with small *k_d_* can obtain good aperture accuracy. However, when *k_d_* is 0.4, due to the value of *k_d_* is too small, the drilling ability of primary drill bit is lower, by contrast thrust force generated by the secondary drill bit rises and fluctuate generated by thrust force increases greatly, which can increase the aperture size. Therefore, step drill can effectively reduce the aperture deviation, choosing an appropriate range of *k_d_* can greatly improve the consistency of the aperture size and is more conducive to the assembly of stacks workpieces.

### 3.4. Effect of Step Drill Structure on the Hole Wall Quality

**Figure 8:**
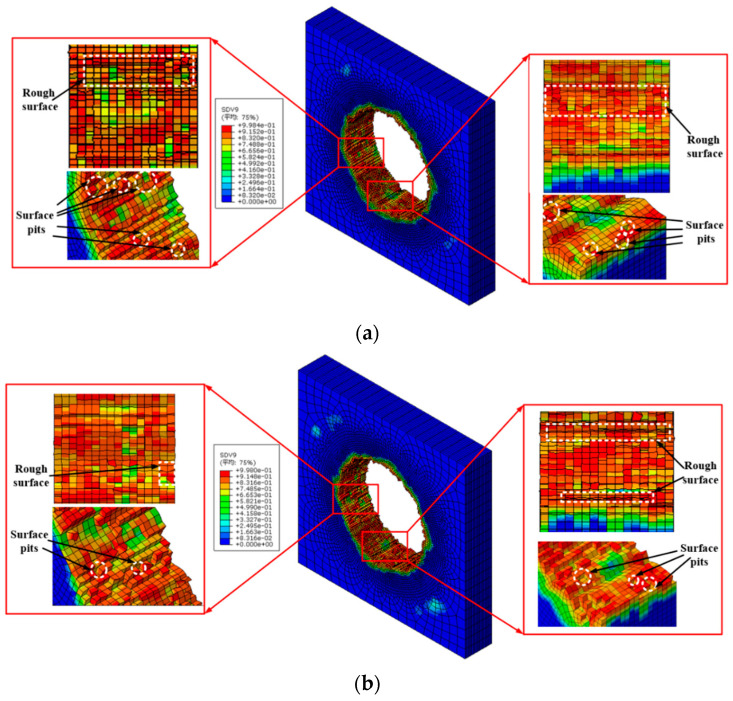

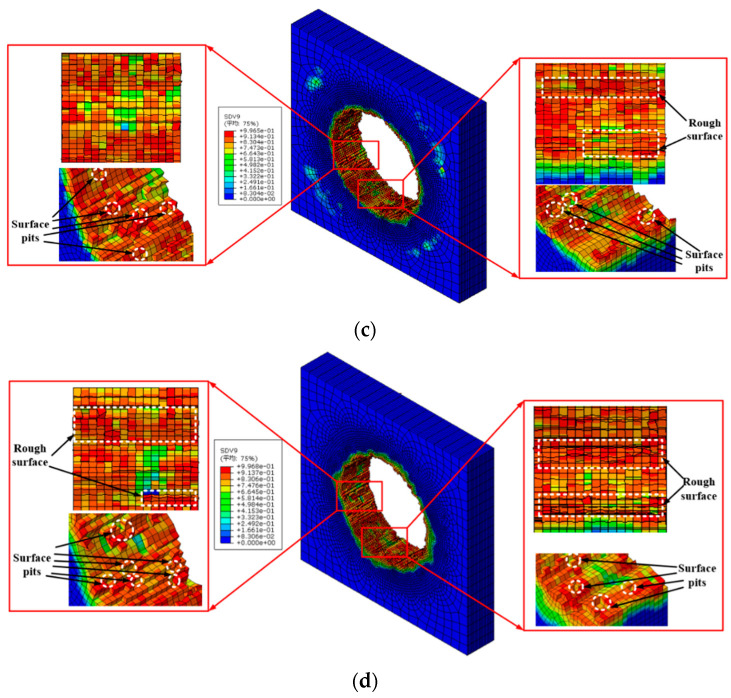
Morphology of CFRP hole wall obtained from simulation model. (**a**) Morphology of hole wall as k_d_ is 0.4; (**b**) Morphology of hole wall as k_d_ is 0.6; (**c**) Morphology of hole wall as k_d_ is 0.8; (**d**) Morphology of hole wall as k_d_ is 1.0.

As drilling of CFRP/Ti stacks, CFRP is the more concentrated area of hole defects. Surface pits are the main manifestation of surface defects in CFRP layer, which are the main reason for the decrease of surface quality during CFRP machining. The morphology of hole wall simulated as drilling of CFRP/Ti stacks with different step drills are shown in Figure 8. SDV9 in Figure 8 represents the damage variable of the CFRP self-defined in the simulation model. Based on the results, it is evident that the quality of CFRP/Ti stacks hole wall drilled with step drill has fewer pit defects, which can effectively improve the surface quality of the hole wall. When *k_d_* is 0.6, there are fewer hole wall surface defects.

The special structure of the step drill can ensure low thrust force and low vibration during the machining process, which can make the drilling process is smoother and the surface quality of the hole wall is better. Besides, the secondary cutting edge of the tool can remove some of the hole wall defects generated by the first stage of the tool. Moreover, the chips generated by Ti can also affect surface quality of the hole, which can scratch the hole wall and cause the quality of the hole wall to deteriorate.

## 4. Conclusions

In this study, a simulation model for drilling of CFRP/Ti stacks is established to investigate the relationship between step drill structure and formation process of stacks hole damage. The conclusions obtained are as follows:

(1) Thrust force and the maximum height of the titanium alloy exit burrs are selected as two indicators to verify the accuracy of the simulation model. The proposed simulation model is found to accurately predicted thrust force and burrs of Ti which are in the maximum error of 15.59% and 3.71% respectively. Based on this result, the reliability of the simulation model results has been verified.
(2) The structure of step drill is confirmed to have significant effect on reducing the height of the titanium alloy burr at hole exit. When the stacking sequence is CFRP to Ti, the height of the titanium alloy burr at hole exit decreases first and then increases with the rising of *k_d_*. When *k_d_* is 0.6, the height of the titanium alloy burr at hole exit is the lower.
(3) When the stacking sequence is CFRP to Ti, the delamination damage of the CFRP in this stacking sequence is less affected by the change in step drill structure. The results show that as drilling from CFRP to Ti, the support of Ti can offset part of the punching on the CFRP by step drill, which makes the delamination factor only increase by 2.57% with *k_d_* increases from 0.4 to 1.0.
(4) The aperture accuracy is related to the structure of step drill. The aperture size decreases first and then increases with the rising of *k_d_*. When *k_d_* is 0.6, the aperture size deviation is the smallest and the aperture accuracy is the best.
(5) The structure of step drill has a certain impact on the quality of the hole wall. Fewer pit defects on the hole wall surface of CFRP layer occurs as drilling with step drill. Especially when *k_d_* is 0.6, the quality of the hole wall surface is better.

## Figures and Tables

**Table 1:** Mechanical properties of CFRP.

Mechanical Property	Value
Density, *ρ* (Kg/m^3^)	1470
Longitudinal modulus, *E*_1_ (GPa)	116
Transverse modulus, *E*_2_ (GPa)	8.5
Transverse modulus, *E*_3_ (GPa)	8.5
Poisson ratio, *υ*_12_	0.32
Poisson ratio, *υ*_13_	0.32
Poisson ratio, *υ*_23_	0.46
Shear modulus in 1–2 plane, *G*_12_ (GPa)	3.26
Shear modulus in 1–3 plane, *G*_13_ (GPa)	3.26
Shear modulus in 2–3 plane, *G*_23_ (GPa)	2.91
Shear strength in 1–2 plane, *S*_12_ (MPa)	79
Shear strength in 1–3 plane, *S*_13_ (MPa)	79
Shear strength in 2–3 plane, *S*_23_ (MPa)	79
Longitudinal tensile strength, *X*_T_ (MPa)	1950
Longitudinal compressive strength, *X*_C_ (MPa)	1480
Transverse tensile strength, *Y*_T_ (MPa)	48
Transverse compressive strength, *Y*_C_ (MPa)	200
Transverse tensile strength, *Z*_T_ (MPa)	48
Transverse compressive strength, *Z*_C_ (MPa)	200

**Table 2:** JC constitutive model parameters and J-C damage criteria parameters.

JC constitutive model	*A* (MPa)	*B* (MPa)	*C*	*n*	*m*
	860	683	0.035	0.47	1
JC damage criteria	*D*_1_	*D*_2_	*D*_3_	*D*_4_	*D*_5_
	−0.09	0.25	−0.5	0.014	3.87

**Table 3:** Properties of interface phase.

Mechanical Property	Value
*K_nn_* (Gpa)	2
*K_ss_* (Gpa)	1.5
*K_tt_* (Gpa)	1.5
$t_{n}^{f}$ (Mpa)	60
$t_{s}^{f}$ (Mpa)	90
$t_{t}^{f}$ (Mpa)	90

**Table 4:** Geometrical parameters of step drills.

	*k_d_*	Point Angle of Primary Drill Bit (°)	Point Angle of Secondary Drill Bit (°)	Tool Diameter (mm)
1	0.4	130	130	9.53
2	0.6	130	130	9.53
3	0.8	130	130	9.53
4	1.0	130	-	9.53

## References

[1] Abidin N.M.Z., Sultan M.T.H., Hua L.S., Basri A.A., Md Shah A.U., Safri S.N.A. (2019). A brief review of computational analysis and experimental models of composite materials for aerospace applications. J. Reinf. Plast. Compos..

[2] Choi J.Y., Jeon J.H., Lyu J.H., Park J., Kim G.Y., Chey S.Y., Quan Y.-J., Bhandari B., Prusty B.G., Ahn S.-H. (2022). Current Applications and Development of Composite Manufacturing Processes for Future Mobility. Int. J. Precis. Eng. Manuf. Green Technol..

[3] Das T.K., Ghosh P., Das N.C. (2019). Preparation, development, outcomes, and application versatility of carbon fiber-based polymer composites: A review. Adv. Compos. Hybrid Mater..

[4] Jones C.E., Norman P.J., Burt G.M., Hill C., Allegri G., Yon J.M., Hamerton I., Trask R.S. (2021). A Route to Sustainable Aviation: A Roadmap for the Realization of Aircraft Components With Electrical and Structural Multifunctionality. IEEE Trans. Transp. Electrif..

[5] Zheng H., Zhang W.J., Li B.W., Zhu J.J., Wang C.H., Song G.J., Wu G.S., Yang X.P., Huang Y.D., Ma L.C. (2022). Recent advances of interphases in carbon fiber-reinforced polymer composites: A review. Compos. Part B Eng..

[6] Xu J., Ji M., Davim J.P., Chen M., El Mansori M., Krishnaraj V. (2020). Comparative study of minimum quantity lubrication and dry drilling of CFRP/titanium stacks using TiAIN and diamond coated drills. Compos. Struct..

[7] Ge J., Chen G., Su Y., Zou Y., Ren C., Qin X., Wang G. (2022). Effect of cooling strategies on performance and mechanism of helical milling of CFRP/Ti-6Al-4 V stacks. Chin. J. Aeronaut..

[8] An Q.L., Zhong B.F., Wang X.F., Zhang H.Z., Sun X.F., Chen M. (2021). Effects of drilling strategies for CFRP/Ti stacks on static mechanical property and fatigue behavior of open-hole CFRP laminates. J. Manuf. Process..

[9] Jia Z.Y., Fu R., Niu B., Qian B.W., Bai Y., Wang F.J. (2016). Novel drill structure for damage reduction in drilling CFRP composites. Int. J. Mach. Tools Manuf..

[10] SenthilKumar M., Prabukarthi A., Krishnaraj V. (2013). Study on Tool Wear and Chip Formation During Drilling Carbon Fiber Reinforced Polymer (CFRP)/Titanium Alloy (Ti_6_Al_4_V) Stacks. Procedia Eng..

[11] Shi R.P., Wang C.Y., Wang X. (2012). Preliminary Study on Carbon Fibre Composites Cutting Technology and Cutting Tools. Mater. Sci. Forum.

[12] Baon F., Sambruno A., Batista M., Fernandez-Vidal S.R., Salguero J. Study of the one-shot drilling of CFRP/Ti_6_Al_4_V stacks with a double tip angle cutting-tool geometry. Proceedings of the International ESAFORM Conference on Material Forming.

[13] Liu L.P., Lian B., Zhou C.G., Duan K.H., Zhu X.M., Xia P.F. (2021). Research on drilling CFRP laminate with a thin woven glass fiber surface layer using plane rake-faced twist drill. Int. J. Adv. Manuf. Technol..

[14] Alonso U., Calamaz M., Girot F., Iriondo E. (2019). Influence of flute number and stepped bit geometry when drilling CFRP/Ti6Al4V stacks. J. Manuf. Process..

[15] Wang F.J., Zhao M., Fu R., Liu X., Qiu S., Yan J.B., Zhang B.Y. (2022). Replaceable drill bit with compound step and sawtooth structures for damages and drilling-cost reduction of CFRP composite. J. Manuf. Process..

[16] Brinksmeier E., Janssen R. (2002). Drilling of multi-layer composite materials consisting of carbon fiber reinforced plastics (CFRP), titanium and aluminum alloys. Cirp Ann. Manuf. Technol..

[17] Leng X.L., Li P.N., Niu Q.L., Qiu X.Y., Li C.P. (2018). Tool wear of step drill drilling CFRP/ TC4 stack. Aerosp. Mater. Technol..

[18] Leng X.L., Li P.N., Niu Q.L., Qiu X.Y., Xu L. (2018). Drilling force and hole quality of step drill drilling of carbon fiber composite. Aerosp. Mater. Technol..

[19] Diaz-Alvarez A., Diaz-Alvarez J., Santiuste C., Miguelez M.H. (2019). Experimental and numerical analysis of the influence of drill point angle when drilling biocomposites. Compos. Struct..

[20] Wang H.T., Huang S.T., Zhang P., Xv L.F. (2018). Finite element analysis on influence which tool structure impose on delamination damage of CFRP/Al stacks. Tool Eng..

[21] Isbilir O., Ghassemieh E. (2013). Numerical investigation of the effects of drill geometry on drilling induced delamination of carbon fiber reinforced composites. Compos. Struct..

[22] Yi Y.P., Xv Z.F., Wang H. (2012). Stiffness degradation methodology for low-velocity impact simulation in composite laminate. Chin. Q. Mech..

[23] Rentsch R., Pecat O., Brinksmeier E. (2011). Macro and micro process modeling of the cutting of carbon fiber reinforced plastics using FEM. Procedia Eng..

[24] Johnson G.R. A Constitutive Model and Data for Metals Subjected to Large Strains, High Strain Rates and High Temperature. Proceedings of the 7th International Symposium on Ballistics.

[25] Ji C.H., Li Y.H., Qin X.D., Zhao Q., Sun D., Jin Y. (2015). 3D FEM simulation of helical milling hole process for titanium alloy Ti-6Al-4V. Int. J. Adv. Manuf. Technol..

[26] Xu J., Lin T., Li L., Ji M., Davim J.P., Geier N., Chen M. (2022). Numerical study of interface damage formation mechanisms in machining CFRP/Ti6Al4V stacks under different cutting sequence strategies. Compos. Struct..

[27] Xu J.Y., El Mansori M. (2016). Cutting Modeling of Hybrid CFRP/Ti Composite with Induced Damage Analysis. Materials.

